# Secretion of and Self-Resistance to the Novel Fibupeptide Antimicrobial Lugdunin by Distinct ABC Transporters in Staphylococcus lugdunensis

**DOI:** 10.1128/AAC.01734-20

**Published:** 2020-12-16

**Authors:** Sophia Krauss, Alexander Zipperer, Sebastian Wirtz, Julian Saur, Martin C. Konnerth, Simon Heilbronner, Benjamin O. Torres Salazar, Stephanie Grond, Bernhard Krismer, Andreas Peschel

**Affiliations:** a Interfaculty Institute of Microbiology and Infection Medicine, Infection Biology, University of Tübingen, Tübingen, Germany; b German Center for Infection Research (DZIF), Tübingen, Germany; c Cluster of Excellence: EXC 2124: Controlling Microbes to Fight Infection, Tübingen, Germany; d Institute of Organic Chemistry, University of Tübingen, Tübingen, Germany

**Keywords:** ABC transporters, *Staphylococcus*, drug resistance mechanisms, natural antimicrobial products

## Abstract

Lugdunin is the first reported nonribosomally synthesized antibiotic from human microbiomes. Its production by the commensal Staphylococcus lugdunensis eliminates the pathogen Staphylococcus aureus from human nasal microbiomes. The cycloheptapeptide lugdunin is the founding member of the new class of fibupeptide antibiotics, which have a novel mode of action and represent promising new antimicrobial agents. How S. lugdunensis releases and achieves producer self-resistance to lugdunin has remained unknown. We report that two ABC transporters encoded upstream of the lugdunin-biosynthetic operon have distinct yet overlapping roles in lugdunin secretion and self-resistance. While deletion of the *lugEF* transporter genes abrogated most of the lugdunin secretion, the *lugGH* transporter genes had a dominant role in resistance. Yet all four genes were required for full-level lugdunin resistance. The small accessory putative membrane protein LugI further contributed to lugdunin release and resistance levels conferred by the ABC transporters. Whereas LugIEFGH also conferred resistance to lugdunin congeners with inverse structures or with amino acid exchange at position 6, they neither affected the susceptibility to a lugdunin variant with an exchange at position 2 nor to other cyclic peptide antimicrobials such as daptomycin or gramicidin S. The obvious selectivity of the resistance mechanism raises hopes that it will not confer cross-resistance to other antimicrobials or to optimized lugdunin derivatives to be used for the prevention and treatment of S. aureus infections.

## Abstract (precis)

Lugdunin is the first reported nonribosomally synthesized antibiotic from human microbiomes. Its production by the commensal Staphylococcus lugdunensis eliminates the pathogen Staphylococcus aureus from human nasal microbiomes. The cycloheptapeptide lugdunin is the founding member of the new class of fibupeptide antibiotics, which have a novel mode of action and represent promising new antimicrobial agents. How S. lugdunensis releases and achieves producer self-resistance to lugdunin has remained unknown.

## INTRODUCTION

The dynamic changes in microbiome composition are governed by multiple antagonistic or mutualistic microbial interactions (1). Several microbiome members achieve fitness benefits in competition with other bacteria through the production of bacteriocins or related antimicrobials (2, 3). The biosynthetic genes for the production of antimicrobials are located in highly variable and often mobile clusters, which usually also include genes conferring self-resistance to the producer strain (4, 5). Such mechanisms can confer resistance to a more or less narrow range of antimicrobials, thus defining the capacity of antimicrobial-producing bacterial strains to tolerate their own compound plus, potentially, those from competitors. The capacity to produce bacteriocins and related molecules has been found to be particularly abundant in microbiome members from nutrient-poor habitats such as the human nose (6). We are only beginning to understand the diversity and relevance of such molecules (7).

We have recently reported that most isolates of Staphylococcus lugdunensis, a colonizer of the human skin and nasal mucosa, produce lugdunin, the founding member of a new class of circular antimicrobial peptides named fibupeptides (8, 9). Lugdunin is synthesized by nonribosomal peptide synthetases and inhibits target bacteria by dissipating their membrane potential, probably in a protonophore-like fashion (9). In addition to its direct antimicrobial activity, lugdunin stimulates human skin cells to produce antibacterial host defense peptides that synergize with lugdunin in the elimination of susceptible microbes (10). Lugdunin-producing S. lugdunensis can eradicate the major human pathogen Staphylococcus aureus, and nasal carriage of S. lugdunensis strongly reduces the rate of nasal colonization by S. aureus (8). The suitability of lugdunin as a potential new drug for S. aureus decolonization and therapy depends also on the risk of resistance development. We found that S. aureus cannot develop spontaneous resistance to lugdunin even after several passages in cultures with increasing subinhibitory concentrations of lugdunin (8). It has remained unclear, though, how S. lugdunensis achieves self-resistance to its product and if potential resistance genes could be mobilized and transferred to S. aureus or other pathogens.

Here, we analyzed the *lugEFGH* genes encoded next to the lugdunin biosynthesis genes and show that the four ABC transporter-encoding genes are necessary and sufficient to confer lugdunin resistance. LugEFGH and the accessory small putative membrane protein LugI were required for both optimal secretion of endogenous lugdunin and resistance to exogenous lugdunin, and even slight changes in lugdunin structure abrogated the capacity of the ABC exporters to protect against these compounds.

## RESULTS

### The lugdunin gene cluster includes 13 genes, many of which encode proteins of unknown functions.

**FIG 1:**
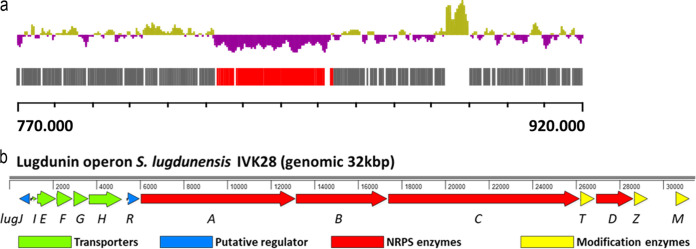
Decreased G+C content (a) and genetic organization (b) of the lugdunin gene cluster. S. lugdunensis IVK28 chromosomal section between nucleotides 770,000 and 920,000 (BioProject accession number PRJNA669000 and GenBank accession number CP063143), along with the encoded open reading frames in red (lugdunin gene cluster *lugJ* to *lugM*) and gray (other genes) and the corresponding G+C content in purple (below average; 26.7% for the lugdunin operon) and green (above average; 33.82% for the entire genome), is shown in panel a. Organization of the lugdunin gene cluster with functional assignment in different colors is shown in panel b. Protein accession numbers are listed in Table S3 in the supplemental material.

The recent identification of the lugdunin gene cluster comprising the biosynthetic *lugABCD* genes and the putative regulator *lugR* (8) prompted us to elucidate the boundaries of the cluster and identify additional genes potentially involved in lugdunin synthesis, export, regulation, and self-resistance. The cluster, plus some of the adjacent genes, has a significantly lower G+C content than the rest of the chromosome (26.7 versus 33.8%, respectively), and the region spanning *lugH* and *lugR* has even less than 24% G+C (Fig. 1a), suggesting that *lugRABCD* plus nine additional genes form the full gene cluster (Fig. 1b). *lugD*, coding for the starter unit in lugdunin biosynthesis, is flanked by the genes encoding LugT, a putative type II thioesterase that may repair stalled peptidyl carrier protein (PCP) domains (11), and LugZ, which is homologous to 4′-phosphopantetheinyl transferases and probably converts apo-PCP to the active holo-form by attachment of the 4-phosphopantetheine cofactor (11). Further downstream, probably forming a separate transcriptional unit, *lugM* encodes a putative monooxygenase, whose role in the biosynthesis process remains unclear.

Upstream of *lugR*, five genes (*lugIEFGH*) form another operon (Fig. 1b). LugI is predicted to encode a 79-amino-acid-long integral membrane protein with two transmembrane helices and no similarity to proteins of known function (Fig. S1 in the supplemental material). LugE and LugG contain conserved Walker motifs probably representing the ATP-binding components of ABC transporter complexes (12). LugF and LugH are related to the integral membrane parts of putative ABC transporters of other *Firmicutes*, with LugF containing 6 and LugH 12 putative transmembrane segments (Fig. S1). According to the canonical architecture of ABC transporter complexes, the four proteins could form two distinct transporters, one as a LugEF homodimer and a second with a LugG homodimer linked to one LugH copy. Upstream of *lugI*, the gene *lugJ* is encoded in opposite direction, which may constitute a second regulator gene in addition to *lugR*. LugJ most likely belongs to the winged-helix type HTH-containing transcriptional regulators. Most antibiotic biosynthetic gene clusters encode proteins conferring self-resistance to the producing strain. Usually, these are either antibiotic-insensitive variants of target proteins, enzymes for the modification of target structures (e.g., rRNAs), or antibiotic exporters (13). None of the genes in the lugdunin cluster seemed to reflect the first two types of self-resistance genes, while the putative ABC transporter genes were regarded as candidates for accomplishing lugdunin secretion and self-resistance and were analyzed further.

### ABC transporters encoded in the *lug* gene cluster mediate lugdunin release and confer resistance to lugdunin.

**FIG 2:**
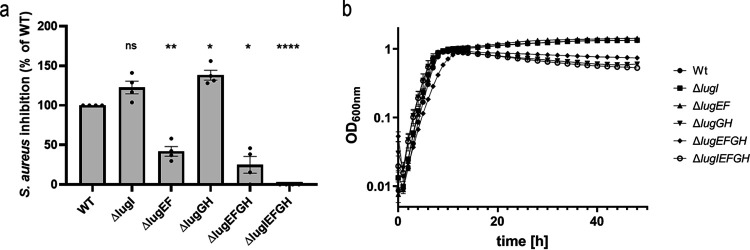
Impact of combinations of deletions of the *lugIEFGH* genes on S. lugdunensis lugdunin secretion (a) and growth (b). (a) Differences in inhibition zone distances around colonies of S. lugdunensis wild type (WT), set to 100%, or mutants with the indicated deletions on agar containing lugdunin-susceptible S. aureus. (b) Growth in broth culture of the strains shown in panel a. Means and SEM of at least 4 (panel a) or 3 (panel b) independent experiments are shown. Significant differences were calculated by one-way ANOVA (Dunnett's multiple-comparison test) (*, *P* ≤ 0.05; **, *P* ≤ 0.01; ***, *P* ≤ 0.001; ****, *P* < 0.0001; ns, not significant).

To analyze a potential role of the ABC transporters in lugdunin export and self-resistance, different combinations of *lugEFGH* and the cotranscribed gene *lugI* were deleted in the lugdunin-producing strain S. lugdunensis IVK28. To avoid polar effects on downstream transcripts, an allelic replacement strategy with no insertion of foreign DNA fragments was used. When inhibition zones around spotted bacterial suspensions with identical diameters of the wild type and mutants on agar containing lugdunin-susceptible S. aureus cells were compared (Fig. 2a), the *lugIEFGH* mutant (Δ*lugIEFGH*) showed no inhibition, indicating that some or all of the five genes are required for lugdunin export. Deletion of only *lugEFGH* strongly reduced but did not abolish lugdunin release. The inhibitory distance was about 25% compared to the wild type (Fig. 2a), suggesting that LugI has a very modest but LugEFGH-independent role in lugdunin release. However, the sole inactivation of *lugI* caused no reduction in lugdunin release. Deletion of *lugEF* had a significant impact on the level of lugdunin export, which was almost as strong as in the Δ*lugEFGH* mutant, indicating that LugEF has a dominant role in lugdunin export. In contrast, the Δ*lugGH* mutant released even slightly larger amounts of lugdunin (about 38%) and exhibited a growth defect in liquid culture compared to the wild type (Fig. 2b), suggesting a role in resistance to lugdunin rather than export. Accordingly, the other *lugGH*-deficient mutant strains Δ*lugEFGH* and Δ*lugIEFGH* displayed similar growth defects (Fig. 2b).

**FIG 3:**
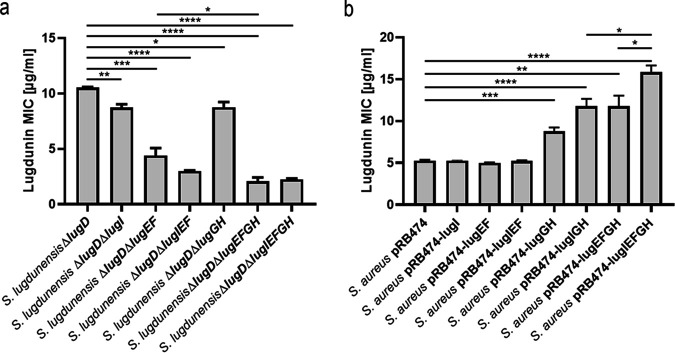
Impact of *lugIEFGH* deletion in the S. lugdunensis Δ*lugD* strain (a) or constitutive expression in S. aureus (b) on lugdunin susceptibility. Means and SEM of at least five independent experiments are shown. Significant differences were calculated by one-way ANOVA (Brown-Forsythe and Welsh) (*, *P* ≤ 0.05; **, *P* ≤ 0.01; ***, *P* ≤ 0.001; ****, *P* < 0.0001).

To investigate the role of LugIEFGH in lugdunin self-resistance, several combinations of the genes were deleted in S. lugdunensis Δ*lugD*, which does not produce lugdunin (8), and the susceptibility of the resulting mutants to lugdunin was analyzed. Deletion of the entire gene set (*lugIEFGH*) strongly decreased the MIC to exogenous lugdunin from 10.5 μg/ml to 2.0 μg/ml, indicating that the genes are involved in producer self-resistance to lugdunin (Fig. 3a). Deletion of either *lugEF* or *lugGH* also led to reduced lugdunin MIC values, indicating that both ABC transporters play a role in lugdunin self-resistance. Deletion of *lugI* led to a decrease of the MIC to the identical level as the *lugGH* deletion. Deletion of *lugEF*, *lugIEF*, or *lugIEFGH* led to a stepwise MIC decrease to the lowest observed level. Δ*lugEFGH*, still expressing *lugI*, showed the same MIC level as the *lugIEFGH* mutant, indicating that although *lugI* deletion has an effect on the overall MIC level, LugI seems to rely on the presence of one of the transporters to modulate lugdunin self-resistance (Fig. 3a). The lugdunin MIC of the S. lugdunensis *lugEFGH* deletion mutant was at the same level as those of a representative panel of nasal S. aureus and Staphylococcus epidermidis strains (2.7 μg/ml on average; Fig. S2), suggesting that there is probably no additional self-resistance system involved.

To confirm the capacity of *lugIEFGH* to confer lugdunin resistance, the genes were cloned in different combinations in the pRB474 vector downstream of a constitutive promoter and introduced into S. aureus N315. *lugGH* expression led to a significantly increased lugdunin MIC (Fig. 3b), which confirms the important contribution of this subset of genes to lugdunin resistance. The additional expression of *lugEF* further raised the resistance of S. aureus to lugdunin, which supports the notion that full lugdunin resistance depends on the presence of all four ABC transporter genes. However, expression of *lugEF* alone did not cause a notable level of resistance. The presence of the entire operon *lugIEFGH* increased the lugdunin MIC to the highest observed level of 15.9 μg/ml, indicating that the small *lugI* also contributes to resistance. When *lugI* was expressed in combination with *lugEF* (pRB474-lugIEF), no increased MIC compared to *lugEF* expression alone was observed. In contrast, *lugI* expression with *lugGH* (pRB474-lugIGH) enhanced the MIC to the same level as *lugEFGH* expression, indicating that LugI might have a supporting effect with LugGH rather than with LugEF. Accordingly, the exclusive expression of *lugI* did not alter the susceptibility to lugdunin. The lugdunin MIC reached in S. aureus pRB474-lugIEFGH was identical to or even higher than that of S. lugdunensis IVK28, probably as a consequence of the high plasmid copy number (Fig. 3b).

### The resistance conferred by the ABC transporters LugIEFGH is largely specific for native lugdunin.

**FIG 4:**
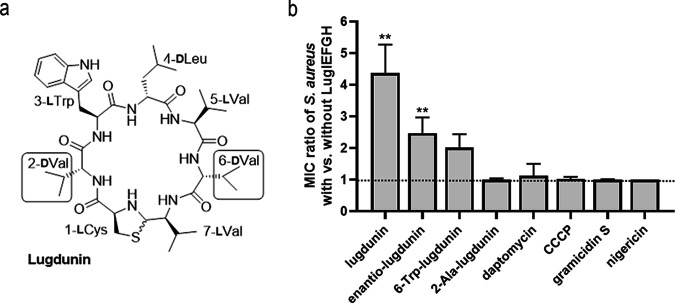
Impact of *lugIEFGH* on S. aureus susceptibility to lugdunin variants and other cyclic peptide antimicrobials. (a) Chemical structure of lugdunin and positions of alterations in derivatives used in panel b. (b) Ratios of MICs elucidated for S. aureus pRB474-lugIEFGH versus S. aureus pRB474. Means ± SEM from at least three independent experiments and significant differences between MICs for the two strains, calculated by Student’s multiple unpaired *t* test (with Holm-Sidak correction) are shown (**, *P* ≤ 0.01). Mean MIC values for all compounds and both strains are listed in Table S3 in the supplemental material.

While some ABC drug exporters have broad substrate specificities, others are highly selective for only certain compounds (14). The *lugIEFGH* genes were assessed for their capacity to protect S. aureus against lugdunin derivatives (see chemical structures 1 to 4 in Fig. S3) and other antimicrobial compounds to elucidate the transporters’ substrate range. The three derivatives enantio-lugdunin, 6-Trp-lugdunin, and 2-Ala-lugdunin were selected because they had similar activities as native lugdunin. 6-Trp-lugdunin was even slightly more active than native lugdunin. Since most other lugdunin derivatives showed no or only residual activity, we could include only the two active versions (9). The constitutive expression of LugIEFGH did not affect the susceptibility of S. aureus to the membrane-active cyclic peptide antibiotics daptomycin and gramicidin S, or to the small nonpeptide protonophores carbonyl cyanide *m*-chlorophenylhydrazone (CCCP) and nigericin, indicating that the resistance mechanism has a strict preference for the structure of lugdunin (Fig. 4a). LugIEFGH also conferred some degree of resistance to the lugdunin enantiomer (enantio-lugdunin), which has the same structure as regular lugdunin but an inverse d-/l-amino acid configuration (8, 9), albeit with a much lower efficacy as to native lugdunin. Similar, though even less pronounced, findings were obtained with 6-Trp-lugdunin, which contains a d-tryptophan at position 6 instead of a d-valine (9). In contrast, 2-Ala-lugdunin (d-alanine instead of d-valine at position 2) (9) had equal antimicrobial activity against S. aureus with or without LugIEFGH, implying that no resistance against the 2-Ala congener was conferred. Thus, LugIEFGH is largely specific for lugdunin in its native structure, and lugdunin alterations at position 2 are less well tolerated by the transporter than alterations at position 6.

## DISCUSSION

**FIG 5:**
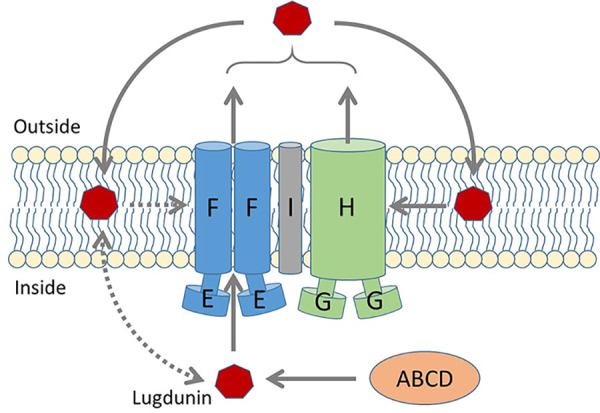
Model for the roles of LugIEFGH in lugdunin secretion and self-resistance. LugEF has a dominant role in lugdunin secretion and a minor role in lugdunin resistance. In contrast, LugGH is mostly responsible for self-resistance, presumably by taking lugdunin up from the membrane bilayer. LugI contributes to secretion and resistance by collaborating with the ABC transporters in a currently unclear fashion. Putative minor lugdunin passageways are shown as dashed arrows. ABCD depicts the intracellular lugdunin-biosynthetic enzymes.

Lugdunin, the first nonribosomally synthesized antibiotic from human microbiomes, has a novel structure and an unusual protonophore-like mode of action, which distinguishes it from most of the antibiotics in clinical use (9). Lugdunin causes proton leakage in synthetic, protein-free membrane vesicles, suggesting that it does not need to target a proteinaceous molecule to exert its antibacterial activity (9). The atypical mode of action raised the question of whether the bacterial producer strains would also use an unusual mechanism to achieve self-resistance to lugdunin. As shown for other cyclic peptides, lugdunin may be able to oligomerize in membranes (15–17), which might also have an influence on its recognition by the lugdunin transporters. We demonstrate that S. lugdunensis uses the four ABC transporter proteins LugEFGH for lugdunin secretion and self-resistance (Fig. 5), which is reminiscent of several other antimicrobial molecule producers (2, 18). The use of two separate ABC transporters for antimicrobial secretion and self-resistance has previously been documented, for instance, for several lantibiotics and other bacteriocins (18). Moreover, the phenol-soluble modulin (PSM) peptides produced by most *Staphylococcus* species are secreted by an ABC transporter complex, which is encoded by four genes most probably forming two separate transporters, PmtAB and PmtCD (19). They confer self-resistance to PSMs and several other membrane-damaging cationic antimicrobial peptides (CAMPs) (20).

The roles of LugEF and LugGH in lugdunin export and self-resistance overlapped to some degree, which is also reminiscent of some bacteriocin-synthetic systems with two separate ABC transporters (21, 22). LugGH had a dominant role in lugdunin resistance in S. aureus, which was even enhanced by the presence of LugI. Accordingly, LugGH had only a weak effect on lugdunin release in S. lugdunensis. Although deletion of *lugGH* in S. lugdunensis had only a minor effect on the MIC compared to *lugEF* deletion, it had a strong impact on the growth and fitness of lugdunin-producing S. lugdunensis, which is in agreement with its capacity to protect the producer against its product. The S. lugdunensis *lugGH* mutant released even slightly more lugdunin than the wild type for unclear reasons, maybe as a consequence of dysregulation of the lugdunin-biosynthetic process in these highly stressed mutant bacteria. This might also be the explanation for the unexpected strong impact of *lugEF* deletion in S. lugdunensis on the MIC, although LugEF does not change the MIC level in S. aureus. As for several other ABC exporters conferring resistance to membrane-active compounds, it can be assumed that LugH takes up its cargo from the membrane bilayer by opening the channel laterally (Fig. 5). In contrast to LugGH, LugEF did not seem to affect the producer’s fitness but had a dominant impact on lugdunin release, probably by acquiring lugdunin from the biosynthetic machinery (LugABCD) in the cytoplasm. Nevertheless, LugEF also contributed to lugdunin resistance, maybe by exporting excess cytoplasmic or membrane-embedded lugdunin (Fig. 5). It is currently not clear if the two transporter systems form indeed a complex together with LugI. It is possible that LugEF may be associated with the biosynthesis machinery formed by LugABCD to directly export newly synthesized lugdunin, which, in addition to the stoichiometry of the LugIEFGH products, remains to be explored.

It remains unclear how LugI may contribute to lugdunin secretion and self-resistance, but it is obvious that its role in resistance depends on the presence of both ABC transporters. Accessory membrane proteins have been described for other ABC transporters, for instance, the S. aureus VraDEH system, which confers resistance to CAMPs. In addition to the ATPase VraD and the integral membrane component VraE, the system includes the small VraH protein, which is required for high-level resistance to gallidermin and daptomycin and has been denoted a “peptide resistance ABC transporter activity modulator” (23), a term also appropriate for LugI. VraH has a similar size and predicted membrane topology as LugI, but no obvious sequence similarity. Accessory integral membrane proteins are also known to complement ABC transporters secreting and conferring producer self-resistance to the lantibiotics epidermin and gallidermin (21, 22).

Only inversion of the lugdunin structure in enantio-lugdunin or a minor change at amino acid position 6 of lugdunin were tolerated by the resistance mechanism, although resistance to these congeners was much less pronounced than for native lugdunin. In contrast, changes at position 2 abrogated the capacity of LugIEFGH to confer resistance completely. The high selectivity distinguishes the lugdunin resistance mechanism from those to other antimicrobial molecules such as PSMs or from multidrug ABC exporters such as Sav1866 (24) or AbcA (25, 26). Slight modifications of lugdunin that maintain or even increase its antimicrobial activity will therefore make it difficult for LugIEFGH to neutralize such variants if they would be developed for clinical use, even if *lugIEFGH* could spread horizontally between different bacterial species. More detailed studies will be necessary to elucidate the molecular basis for the selectivity and elucidate if and which mutations in the self-resistance proteins might alter or broaden its preferences for peptide cargo.

LugIEFGH has never been found outside the *lug* operon of S. lugdunensis, neither in S. aureus nor other nasal microbiome members. Only a few members of the *Bacillales* order, mainly from the environmental or intestinal bacterial genera *Salinicoccus*, *Planococcus*, *Exiguobacterium*, or *Gracilibacillus*, harbor homologs of the *lugIEFGH* cluster, albeit without the lugdunin biosynthesis genes. Additionally, Streptococcus mutans genomes encode an ABC transporter with homology to LugGH, but lack LugI or LugEF homologs. Despite its lower G+C content, the *lug* gene cluster does not seem to constitute a promiscuous genetic element, which may restrict its mobility among species other than S. lugdunensis.

## MATERIALS AND METHODS

### Strains and growth conditions.

The *Staphylococcus* strains used in this study were S. aureus N315, S. aureus USA300 LAC, and S. lugdunensis IVK28. Further strains used for MIC determination were S. aureus N315 with plasmids pRB474, pRB474-lugI, pRB474-lugEF, pRB474-lugIEF, pRB474-lugGH, pRB474-lugIGH, pRB474-lugEFGH, and pRB474-lugIEFGH. The construction of the plasmids is described below. Escherichia coli DC10B was used as the cloning host for further transformation in S. aureus N315 (expression of transporter genes) or S. aureus PS187 for subsequent phage transduction into S. lugdunensis IVK28 (27).

Basic medium (BM; 1% soy peptone A3 [Organotechnie SAS, France], 0.5% Ohly Kat yeast extract [Deutsche Hefewerke GmbH, Germany], 0.5% NaCl, 0.1% glucose, and 0.1% K_2_HPO_4_, pH 7.2) was used as the standard growth medium and for MIC determinations. If necessary, antibiotic was used at a concentration of 10 μg ml^−1^ for chloramphenicol. E. coli transformants were grown in lysogeny broth (LB; Lennox) medium (1% tryptone, 0.5% yeast extract, and 0.5% NaCl; Carl Roth GmbH, Germany) supplemented with 100 μg ml^−1^ ampicillin or corresponding LB agar.

To analyze growth curves, strains were grown overnight in BM with suitable antibiotics under continuous shaking at 37°C. Each strain was adjusted to an optical density at 600 nm (OD_600_) of 1 in Mueller-Hinton broth (MHB), and 2.5 μl of the bacterial stock solutions were pipetted to 500 μl MHB in a 48-well microtiter plate. The plates were incubated for 48 h under continuous shaking in a microplate reader, and the OD_600_ was measured every 15 minutes.

### Synthetic lugdunin congeners and control compounds.

All synthetic lugdunin derivatives were synthesized as described elsewhere (9). Daptomycin (Cubicin) was purchased from MSD Sharp & Dohme GmbH (Haar, Germany); CCCP, gramicidin S, and nigericin were obtained from Sigma-Aldrich (now Merck, Germany).

### Generation of S. lugdunensis IVK28 knockout mutants.

DNA manipulation, isolation of plasmid DNA, and transformation of E. coli were performed by use of standard procedures. Enzymes for molecular cloning were obtained from Thermo Fisher Scientific and New England Biolabs. For the generation of knockout mutants, the temperature-sensitive shuttle vector pBASE6 was used, and mutants were generated by allelic replacement as described previously (28). Flanking regions of the genes to be deleted were amplified by PCR (Table 1) and ligated to shuttle vector pBASE6 after digestion with suitable restriction enzymes. Cloning was performed in E. coli DC10B from where sequence-verified plasmids were transferred to S. aureus PS187 by electroporation. Phage ϕ187 was used for transduction of S. lugdunensis IVK28 as described elsewhere (27). Mutations in S. lugdunensis were confirmed by PCR amplification of the entire *lugJIEFGH* region with control primers and analysis of the fragment sizes in comparison to the wild type. For the construction of the *lugIGH* mutant, the confirmed *lugGH* mutant was transduced with the plasmid for *lugI* deletion, and the second deletion was performed in the Δ*lugGH* background.

**TABLE 1:** Primers used for construction of S. lugdunensis IVK28 mutants and their verification^*a*^

Primer	Sequence (5′–3′)	Assignment
lugI K.O._forw1_SacI	aaa**gagctc**cgttctccacaattctc	Deletion of *lugI* (3′ of *lugJ*)
lugI K.O._rev1_NcoI	ccct**ccatgg**tcattattgataatgataatg	Deletion of *lugI* (5′ of *lugJ*)
lugI K.O._forw2_NcoI	tgat**ccatgg**aaggaggctaataaaaattgatcg	Deletion of *lugI* (5′ of *lugE*)
lugI K.O._rev2_BglII	aat**agatct**ctcatatatcagacaccaactct	Deletion of *lugI* (3′ of *lugE*)
lugJ SacI	aaa**gagctc**cgtcgttctccacaattc	Deletion of *lugIEF* or *lugIEFGH* (3′ of *lugJ*)
lugJ Acc65I u	tatc**ggtacc**cattttcaccctccattatc	Deletion of *lugIEF* or *lugIEFGH* (5′ of *lugJ*)
lugG Acc65I d	agt**ggtacc**cttacattagctgaaagcc	Deletion of *lugEF* or *lugIEF* (5′ of *lugG*)
lugG BglII	gctaagt**agatct**catataccaaatagcca	Deletion of *lugEF* or *lugIEF* (3′ of *lugG*)
lugJI SacI	cca**gagctc**ctaggattaacttgagagg	Deletion of *lugEF* or *lugEFGH* (3′ of *lugJ*)
lugJI Acc65I u	cct**ggtacc**ccaatacactctccctctga	Deletion of *lugEF* or *lugEFGH* (3′ of *lugI*)
lugF SacI	tta**gagctc**cacatattcttgatgatgc	Deletion of *lugGH* (5′ of *lugF*)
lugF Acc65I u	gata**ggtacc**taacacctttatcagaacc	Deletion of *lugGH* (3′ of *lugF*)
lugR Acc65I d	acaa**ggtacc**tgtagtataaaatccac	Deletion of *lugGH*, *lugEFGH*, or *lugIEFGH* (5′ of *lugR*)
lugR BglII	ctt**agatct**tttcagttatcacaacagg	Deletion of *lugGH*, *lugEFGH*, or *lugIEFGH* (3′ of *lugR*)
lugJ region down	gttttggtacctgtacatggtggtggc	5′ of *lugJ* (control)
lugR region up	cttagatcttttcagttatcacaacagg	3′ of *lugR* (control)

a Restriction sites used for cloning are indicated as bold letters.

### Expression of ABC transporter genes in S. aureus N315.

The transporters of S. lugdunensis IVK28 were cloned in pRB474 as follows. For the *lugEF* construct, the primers ABC1-down and ABC2-up (Table 2) were used to amplify *lugEF*, and the primers ABC regulator forw and ABC2-up were used to amplify *lugIEF.* To express only *lugI*, the gene was amplified with primers ABC regulator forw and lugI rev (SacI). *lugGH* was generated with the primers ABC3-down and ABC4-up. For the generation of the *lugIGH* construct, the plasmid pRB474-lugGH was digested with PstI and treated with alkaline phosphatase. Here, *lugI* was amplified with the primers ABC regulator forw and lugI rev (Pst), digested with PstI, and ligated into the PstI-digested pRB474-lugGH. The correct orientation of *lugI* in front of *lugGH* was confirmed by sequencing. *lugEFGH* was generated with the primers ABC1-down and ABC4-up. The PCR fragment for *lugIEFGH* was amplified with the primers ABC regulator forw and ABC4-up. All PCR products and plasmid pRB474 were digested with PstI and SacI to ligate the PCR fragments into pRB474. The resulting constructs pRB474-lugI, pRB474-lugEF, pRB474-lugIEF, pRB474-lugGH, pRB474-lugIGH, pRB474-lugEFGH, and pRB474-lugIEFGH were transferred into E. coli DC10B (29) and subsequently into S. aureus N315.

**TABLE 2:** Primers used for construction of transporter expression vectors^*a*^

Primer (restriction site)	Sequence (5′–3′)	Amplified gene
ABC1-down (PstI)	ggacctatt**ctgcag**ttgattattggaagga	5′ of *lugE*
ABC3-down (PstI)	tgcat**ctgcag**tcattatcaagaaattc	3′ of *lugF*
ABC2-up (SacI)	tat**gagctc**ttagaatttcttgataatgact	5′ of *lugG*
ABC4-up (SacI)	tgt**gagctc**atcttctaataataag	3′ of *lugH*
ABC regulator forw (PstI)	atgta**ctgcag**cattatcattatcaataatg	5′ of *lugI*
lugI rev (SacI)	cattttattc**gagctc**ttaatctcgatc	3′ of *lugI*
lugI rev (Pst)	cattttattc**ctgcag**ttaatctcgatc	3′ of *lugI*

a Restriction sites used for cloning are indicated as bold letters.

### Analysis of lugdunin secretion.

To analyze the capacity of S. lugdunensis IVK28 and its isogenic mutants to export lugdunin, an S. aureus inhibition assay was performed. S. aureus USA300 LAC was grown overnight in BM, and BM agar, cooled down to 50°C after autoclaving, was inoculated to a final OD of 0.00125 with this overnight culture. From this suspension, defined 15-ml agar plates with 8.4 cm diameter were poured. S. lugdunensis strains were grown overnight in BM, centrifuged, and washed in 1/10 volume phosphate-buffered saline (PBS) to remove residual cell-associated lugdunin. After a second centrifugation step, cultures were adjusted to an OD_600_ of 20, and 10 μl of the suspensions were spotted on the solidified BM agar plates containing S. aureus. After drying of the spots, the plates were incubated at 37°C for 24 h, and inhibition zones were photographed and analyzed with ImageJ software (version 1.8.0_112). For each experiment, all strains to be analyzed were spotted on the same agar plate, and the inhibition zone, defined as the distance between the S. lugdunensis IVK28 colony and the growing S. aureus cells, was defined as 100%.

### MIC determination.

Strains used for MIC determinations were grown overnight in BM, with chloramphenicol for plasmid-containing strains, under continuous shaking at 37°C. Each strain was adjusted to OD_600_ of 0.0625 in BM. The antimicrobial molecule stock solutions were serially diluted in BM in 96-well microtiter plates. Each well with 100 μl medium, and chloramphenicol, if required, was inoculated with 2 μl of the OD_600_ of 0.0625 bacterial stock solution. The plates were incubated at 37°C for 24 h under continuous shaking (160 rpm). The OD_600_ of each well was measured with a microplate reader, and the concentration leading to a 75% growth reduction was calculated and defined as the MIC value.

### Statistics.

Statistical analyses were performed using GraphPad Prism 8.01. One-way analysis of variance (ANOVA) was used to compare MIC levels of individual strains against the reference strain, and *t* tests were used for the comparison of MIC levels against various compounds with or without transporter genes.

### Data availability.

Data for S. lugdunensis strain IVK28 were deposited in BioProject under accession no. PRJNA669000 and GenBank accession number CP063143.

## Supplementary Material

Supplemental file 1
