# Supplementary material for: Secretion of and Self-Resistance to the Novel Fibupeptide Antimicrobial Lugdunin by Distinct ABC Transporters in Staphylococcus lugdunensis
Source: Antimicrob Agents Chemother. 2020 Dec 16;65(1):e01734-20. doi: 10.1128/AAC.01734-20 (PMC7927808; doi:10.1128/AAC.01734-20)
Supplement: Supplemental file 1 [file AAC.01734-20-s0001.pdf]

Supplementary information

Protein	Accession number
LugJ	WP_002460039.1
LugI	WP_002460038.1
LugE	WP_002478838.1
LugF	WP_002478839.1
LugG	WP_002460034.1
LugH	WP_002460033.1
LugR	WP_002460032.1
LugA	WP_002492248.1
LugB	WP_081094477.1
LugC	WP_037540567.1
LugT	WP_002460022.1
LugD	WP_002478846.1
LugZ	WP_002478847.1
LugM	WP_002492211.1

Table S1: Accession numbers of the proteins involved in lugdunin biosynthesis, transport and putative regulation

Compound	MIC *S. aureus* pRB474	MIC *S. aureus* pRB474-lugIEFGH	Ratio
lugdunin	8.15	33.68	4.13
Enantio-lugdunin	31.3	69.75	2.23
6-Trp-lugdunin	16.43	31.05	1.89
2-Ala-lugdunin	40.17	39.93	0.99
daptomycin	2.17	2.17	1
CCCP	0.63	0.64	1.02
gramicidin S	2.75	2.74	1.00
nigericin	0.25	0.25	1.00

Table S2: Mean MIC values in BM-medium of all tested compounds for *S. aureus* pRB474 and pRB474-lugIEFGH

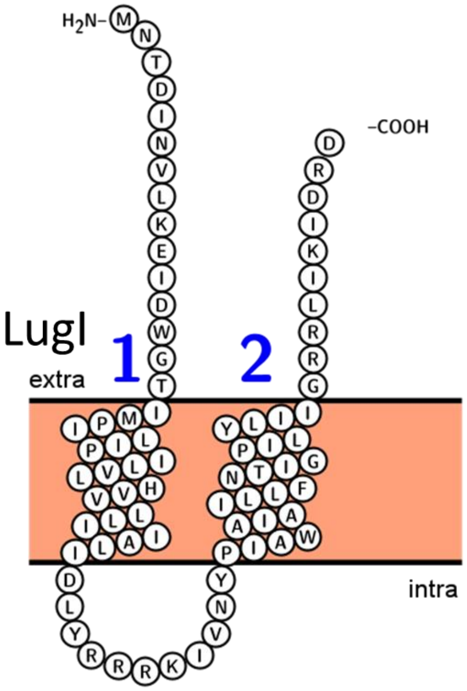
LugI
1
2
extra
intra
H2N–
–COOH

8

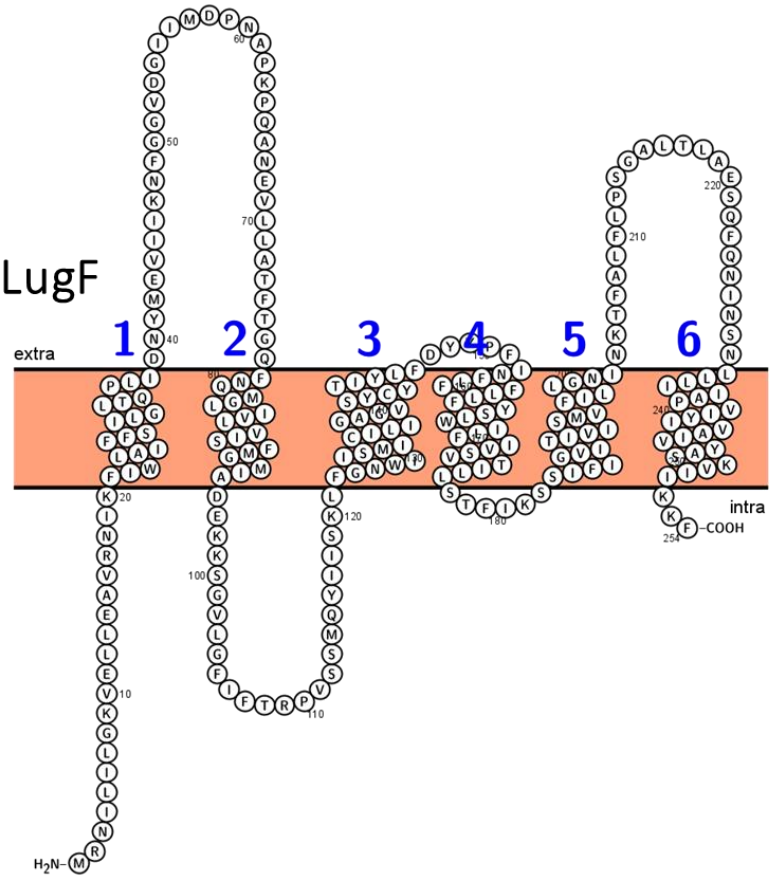
LugF
1
2
3
4
5
6
extra
intra
H2N–
–COOH

9

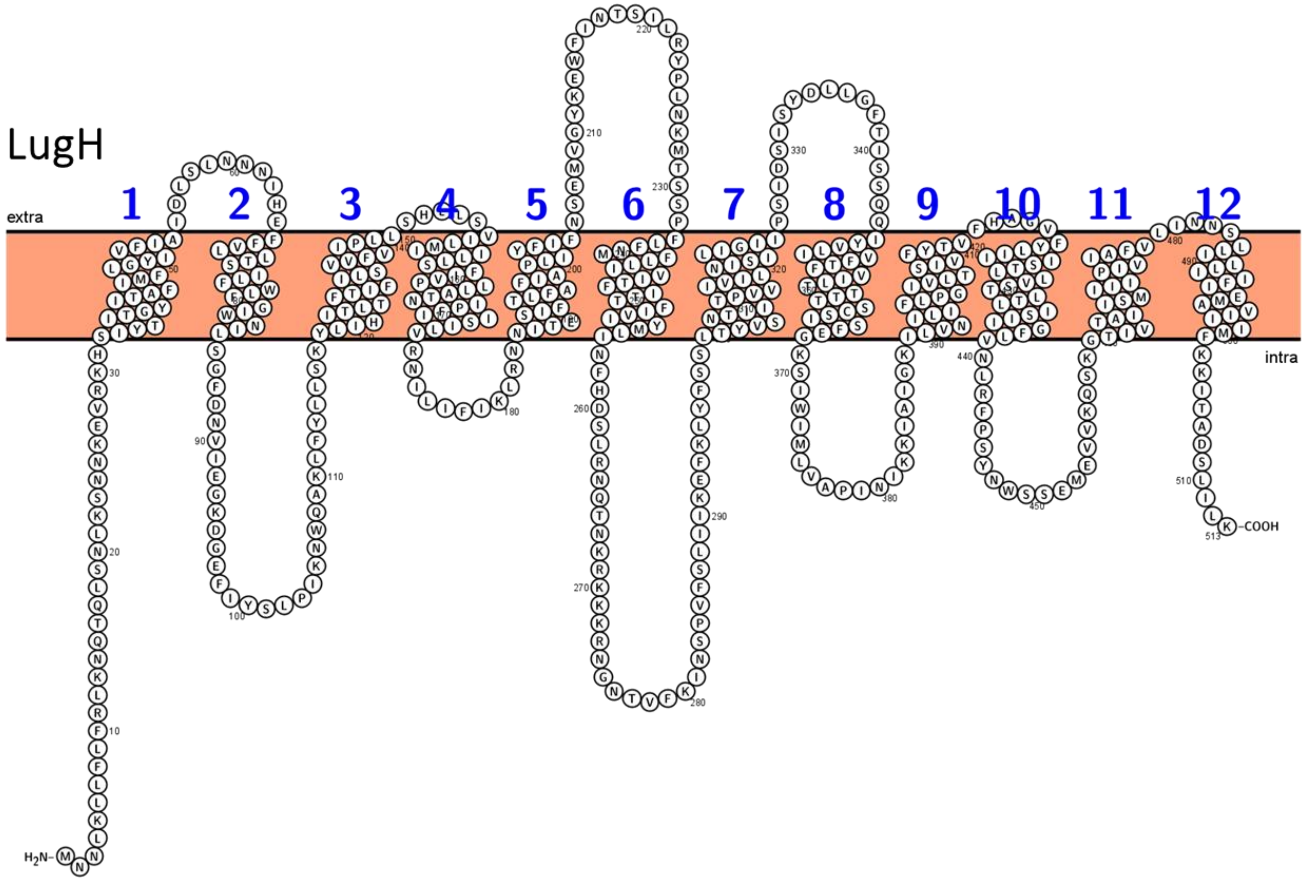

Fig. S1: Predicted transmembrane topologies of LugI, LugF, and LugH. The transmembrane topology for LugI, LugF, and LugH was predicted with the help of PROTTER (version 1.0; http://wlab.ethz.ch/protter/start/) (1).

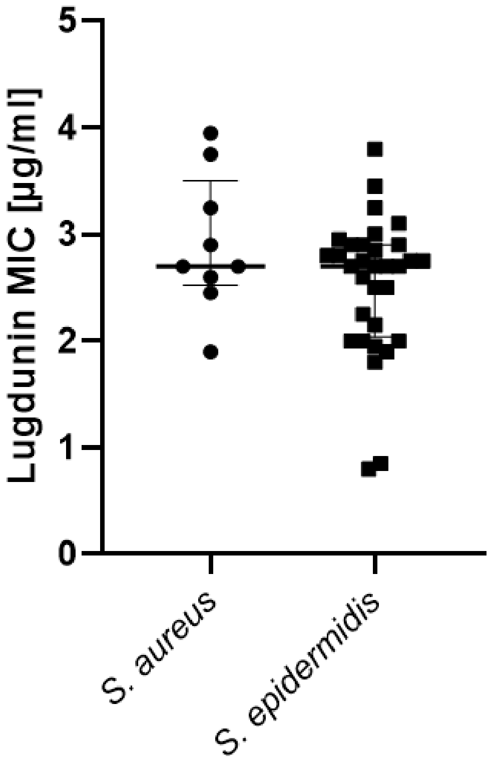

Fig. S2: Comparison of lugdunin susceptibility of individual nasal *S. aureus* and *S. epidermidis* isolates. The MIC of lugdunin was tested against representative nasal *S. aureus* and *S. epidermidis* isolates. Shown are the medians with interquartile range for the two groups.

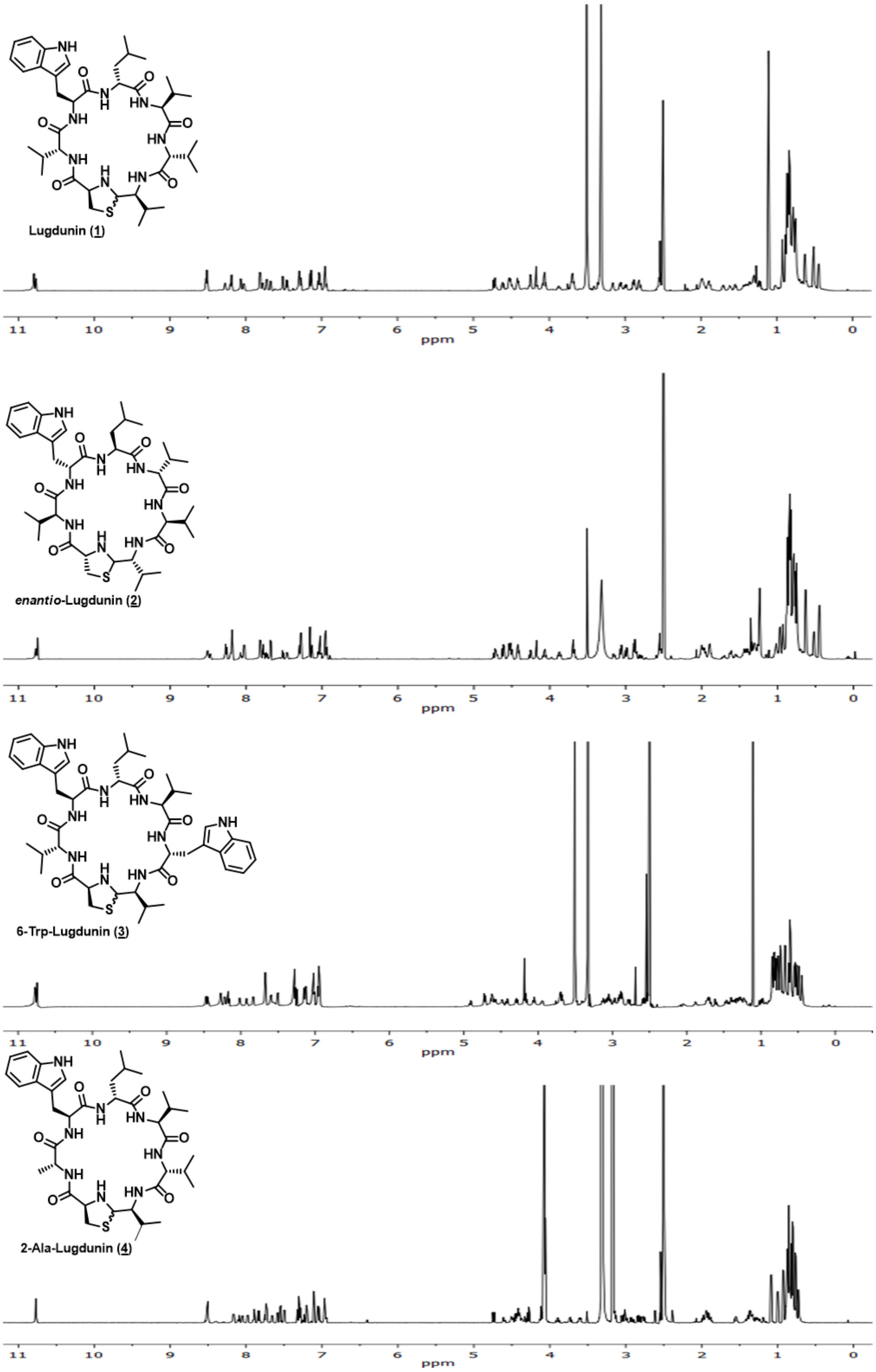

Fig. S3: Chemical structural formula of synthetic lugdunin analogues **1** – **4** and ^{1}H NMR spectra thereof (d_6-dmso, 700 MHz, 303K).

Literature

1. Omasits U, Ahrens CH, Muller S, Wollscheid B. 2014. Protter: interactive protein feature visualization and integration with experimental proteomic data. Bioinformatics 30:884-6.
